# Norwegian Coronavirus Disease 2019 (NO COVID-19) Pragmatic Open label Study to assess early use of hydroxychloroquine sulphate in moderately severe hospitalised patients with coronavirus disease 2019: A structured summary of a study protocol for a randomised controlled trial

**DOI:** 10.1186/s13063-020-04420-0

**Published:** 2020-06-05

**Authors:** Magnus Nakrem Lyngbakken, Jan-Erik Berdal, Arne Eskesen, Dag Kvale, Inge Christoffer Olsen, Anbjørg Rangberg, Christine Monceyron Jonassen, Torbjørn Omland, Helge Røsjø, Olav Dalgard

**Affiliations:** 1 Division of Medicine, Akershus University Hospital, Lørenskog, Norway; 2 Institute of Clinical Medicine, Faculty of Medicine, University of Oslo, Oslo, Norway; 3 Department of Infectious Diseases, Division of Medicine, Akershus University Hospital, Lørenskog, Norway; 4 Department of Infectious Diseases, Oslo University Hospital, Oslo, Norway; 5 Department of Research Support for Clinical Trials, Oslo University Hospital, Oslo, Norway; 6 Center for Laboratory Medicine, Østfold Hospital Trust, Grålum, Norway; 7 Division of Research and Innovation, Akershus University Hospital, Lørenskog, Norway

**Keywords:** COVID-19, Randomized controlled trial, Protocol, Chloroquine, Pragmatic trial, Polymerase chain reaction, Viral load, Oropharyngeal sampling

## Abstract

**Objectives:**

The hypothesis of the study is that treatment with hydroxychloroquine sulphate in hospitalised patients with coronavirus disease 2019 (Covid-19) is safe and will accelerate the virological clearance rate for patients with moderately severe acute respiratory syndrome coronavirus 2 (SARS-CoV-2) when compared to standard care. Furthermore, we hypothesize that early treatment with hydroxychloroquine sulphate is associated with more rapid resolve of clinical symptoms as assessed by the National Early Warning Score 2 (NEWS2), decreased admission rate to intensive care units and mortality, and improvement in protein biomarker profiles (C-reactive protein, markers of renal and hepatic injury, and established cardiac biomarkers like cardiac troponin and B-type natriuretic peptide).

**Trial design:**

The study is a two-arm, open label, pragmatic randomised controlled group sequential adaptive trial designed to assess the effect on viral loads and clinical outcome of hydroxychloroquine sulphate therapy in addition to standard care compared to standard care alone in patients with established Covid-19. By utilizing resources already paid for by the hospitals (physicians and nurses in daily clinical practice), this pragmatic trial can include a larger number of patients over a short period of time and at a lower cost than studies utilizing traditional randomized controlled trial designs with an external study organization. The pragmatic approach will enable swift initiation of randomisation and allocation to treatment.

**Participants:**

Patients will be recruited from all inpatients at Akershus University Hospital, Lørenskog, Norway. Electronic real-time surveillance of laboratory reports from the Department of Microbiology will be examined regularly for SARS-CoV-2 positive subjects. All of the following conditions must apply to the prospective patient at screening prior to inclusion: (1) Hospitalisation; (2) Adults 18 years or older; (3) Moderately severe Covid-19 disease (NEWS2 of 6 or less); (4) SARS-CoV-2 positive nasopharyngeal swab; (5) Expected time of hospitalisation > 48 hours; and (6) Signed informed consent must be obtained and documented according to Good Clinical Practice guidelines of the International Conference on Harmonization, and national/local regulations. Patients will be excluded from participation in the study if they meet any of the following criteria: (1) Requiring intensive care unit admission at screening; (2) History of psoriasis; (3) Known adverse reaction to hydroxychloroquine sulphate; (4) Pregnancy; or (5) Prolonged corrected QT interval (>450 ms). Clinical data, including standard hospital biochemistry, medical therapy, vital signs, NEWS2, and microbiology results (including blood culture results and reverse transcriptase polymerase chain reaction [RT-PCR] for other upper airway viruses), will be automatically extracted from the hospital electronic records and merged with the study specific database.

**Intervention and comparator:**

Included patients will be randomised in a 1:1 ratio to (1) standard care with the addition of 400 mg hydroxychloroquine sulphate (Plaquenil^TM^) twice daily for seven days or (2) standard care alone.

**Main outcomes:**

The primary endpoint of the study is the rate of decline in SARS-CoV-2 viral load in oropharyngeal samples as assessed by RT-PCR in samples collected at baseline, 48 and 96 hours after randomization and administration of drug for the intervention arm. Secondary endpoints include change in NEWS2 at 96 hours after randomisation, admission to intensive care unit, mortality (in-hospital, and at 30 and 90 days), duration of hospital admission, clinical status on a 7-point ordinal scale 14 days after randomization ([1] Death [2] Hospitalised, on invasive mechanical ventilation or extracorporeal membrane oxygenation [3] Hospitalised, on non-invasive ventilation or high flow oxygen devices [4] Hospitalized, requiring supplemental oxygen [5] Hospitalised, not requiring supplemental oxygen [6] Not hospitalized, but unable to resume normal activities [7] Not hospitalised, with resumption of normal activities), and improvement in protein biomarker profiles (C-reactive protein, markers of renal and hepatic injury, and established cardiac biomarkers like cardiac troponin and B-type natriuretic peptide) at 96 hours after randomization.

**Randomisation:**

Eligible patients will be allocated in a 1:1 ratio, using a computer randomisation procedure. The allocation sequence has been prepared by an independent statistician.

**Blinding (masking):**

Open label randomised controlled pragmatic trial without blinding, no active or placebo control. The virologist assessing viral load in the oropharyngeal samples and the statistician responsible for analysis of the data will be blinded to the treatment allocation for the statistical analyses.

**Numbers to be randomized (sample size):**

This is a group sequential adaptive trial where analyses are planned after 51, 101, 151 and 202 completed patients, with a maximum sample size of 202 patients (101 patients allocated to intervention and standard care and 101 patients allocated to standard care alone).

**Trial Status:**

Protocol version 1.3 (March 26, 2020). Recruitment of first patient on March 26, 2020, and 51 patients were included as per April 28, 2020. Study recruitment is anticipated to be completed by July 2020.

**Trial registration:**

ClinicalTrials.gov number, NCT04316377. Trial registered March 20, 2020.

**Full protocol:**

The full protocol is attached as an additional file, accessible from the Trials website (Additional file 1). In the interest in expediting dissemination of this material, the familiar formatting has been eliminated; this Letter serves as a summary of the key elements of the full protocol.

## Supplementary information

**Additional file 1.** Full study protocol.

## Data Availability

The final trial dataset will be made available to the trial statistician at the Research Support Services, Clinical Trial Unit, Oslo University Hospital, Oslo, Norway, who will perform trial statistical analyses according to the predefined statistical analysis plan.

