# Supplementary material for: Norwegian Coronavirus Disease 2019 (NO COVID-19) Pragmatic Open label Study to assess early use of hydroxychloroquine sulphate in moderately severe hospitalised patients with coronavirus disease 2019: A structured summary of a study protocol for a randomised controlled trial
Source: Trials. 2020 Jun 5;21:485. doi: 10.1186/s13063-020-04420-0 (PMC7273378; doi:10.1186/s13063-020-04420-0)
Supplement: Supplementary file 1 — Additional file 1. Full study protocol. [file 13063_2020_4420_MOESM1_ESM.pdf]

NORWEGIAN CORONAVIRUS DISEASE 2019 (NO COVID-19) STUDY: AN OPEN LABELED RANDOMIZED CONTROLLED PRAGMATIC TRIAL TO EVALUATE THE ANTIVIRAL EFFECT OF CHLOROQUINE IN ADULT PATIENTS WITH SARS-COV-2 INFECTION

Protocol Identification Number: Ahus-NO-COVID-19

EudraCT Number: 2020-001010-38

SPONSOR: **Akershus University Hospital**

Head of Department / Associate Professor Jan Erik Berdal
Department of Infectious Diseases

Akershus University Hospital

Sykehusveien 25

1478 Lørenskog, Norway
Tel: 67 96 00 00

E-mail: jan.erik.berdal@medisin.uio.no

PROTOCOL VERSION NO. 1.3 – 26.03.20

CONTACT DETAILS

**Sponsor:**	**Head of Department / Associate Professor Jan Erik Berdal, MD PhD**<br>Department of Infectious Diseases, Division of Medicine<br>Akershus University Hospital<br>Sykehusveien 25<br>1478 Lørenskog, Norway<br>Tel: +47 48 20 58 17<br>E-mail: jan.erik.berdal@medisin.uio.no
**Principal Investigator:**	**Professor Olav Dalgard, MD PhD**<br>Department of Infectious Diseases, Division of Medicine<br>Akershus University Hospital<br>Sykehusveien 25<br>1478 Lørenskog, Norway<br>Tel: +47 92 61 68 00<br>E-mail: odalgard@uio.no
**Coordinating Investigator:**	**Postdoctoral Fellow Magnus Lyngbakken, MD PhD**<br>Division of Medicine<br>Akershus University Hospital<br>1478 Lørenskog, Norway<br>Tel: +47 93 40 88 37<br>E-mail: magnus.lyngbakken@medisin.uio.no
**Investigator:**	**Head of Division Helge Røsjø, MD PhD**<br>Division of Research and Innovation<br>Akershus University Hospital<br>1478 Lørenskog, Norway<br>Tel: +47 91 54 58 64<br>E-mail: helge.rosjo@medisin.uio.no
**Investigator:**	**Professor Torbjørn Omland, MD PhD MPH**<br>Division of Medicine<br>Akershus University Hospital<br>1478 Lørenskog, Norway<br>Tel: +47 40 10 70 50<br>E-mail: torbjorn.omland@medisin.uio.no
**Investigator:**	**Professor Christine Jonassen, PhD**<br>Center for Laboratory Medicine, Østfold Hospital Trust/ Faculty of Chemistry, Biotechnology and Food Science, Norwegian University of Life Sciences (NMBU)<br>Postboks 300, 1714 Grålum<br>Tel: +47 90 73 45 57<br>E-mail: chrjon@so-hf.no

Investigator: **Professor Dag Kvale, MD PhD**
Institute of Clinical Medicine
University of Oslo, Norway
Tel: +47 22 85 05 46
E-mail: dag.kvale@medisin.uio.no

Investigator: **Senior Consultant Arne Eskesen, MD**
Department of Infectious Diseases, Division of Medicine
Akershus University Hospital
1478 Lørenskog, Norway
Tel: +47 47 90 38 35
E-mail: Arne.N.Eskesen@ahus.no

Monitor: **Clinical Trial Unit**
Oslo University Hospital HF
0424 Oslo, Norway
Tel : +47 23 06 60 34

Statistician: **Inge Christoffer Olsen, PhD**
Clinical Trial Unit
Oslo University Hospital HF
0424 Oslo, Norway
Tel : +47 23 06 60 34
E-mail: inge.christoffer.olsen@gmail.com

SIGNATURE PAGE

Title	Norwegian Coronavirus Disease 2019 (NO COVID-19) Study: An open labeled randomized controlled pragmatic trial to evaluate the antiviral effect of chloroquine in adult patients with SARS-CoV-2 infection
Protocol ID:	NO COVID-19
EudraCT no:	2020-001010-38

I hereby declare that I will conduct the study in compliance with the Protocol, ICH GCP and the applicable regulatory requirements:

Name	Title	Role	Signature	Date
Jan Erik Berdal	Head of Department<br>Associate Professor	Sponsor's representative		
Olav Dalgard	Professor	Principal investigator		

SIGNATURE PAGE SITE INVESTIGATORS

Title	Norwegian Coronavirus Disease 2019 (NO COVID-19) Study: An open labeled randomized controlled pragmatic trial to evaluate the antiviral effect of chloroquine in adult patients with SARS-CoV-2 infection
Protocol ID:	NO-COVID-19
EudraCT no:	

I hereby declare that I will conduct the study in compliance with the Protocol, ICH GCP and the applicable regulatory requirements:

Name	Title	Signature	Date

PROTOCOL SYNOPSIS

Norwegian Coronavirus Disease 2019 (NO COVID-19) Study: An open labeled randomized controlled pragmatic trial to evaluate the antiviral effect of chloroquine in adult patients with SARS-CoV-2 infection

Sponsor	Akershus University Hospital HF
Phase and study type	Phase 4, treatment trial
Investigational Medical Product (IMP) (including active comparator and placebo) :	Hydroxychloroquine sulphate<br><br>No active or placebo control
Centers:	Single center during initial phase and with possible extension to other Norwegian sites pending result from the initial phase. Extension will be according to separate submissions to ethics committee and other regulatory bodies from new centers.
Study Period:	Estimated date of first patient enrolled: 23.03.20<br><br>Anticipated recruitment period: 6 months<br><br>Estimated date of last patient completed: 01.04.21<br><br>Estimated study termination: 23.03.25
Treatment Duration:	Seven days
Follow-up:	Until discharge from the hospital, at 30 and 90 days.
Objectives	Main objective:<br><br>To assess whether treatment with hydroxychloroquine in patients with COVID-19 will increase the decline rate of SARS-CoV-2 in the oropharynx<br><br>Secondary objectives:<br><br>To assess the clinical benefit of chloroquine treatment on<br><br>• National Early Warning Score (NEWS)<br><br>• Risk of clinical deterioration and mortality<br><br>• Biomarker profile

Endpoints:

Primary endpoint:

- Rate of decline in SARS-CoV-2 viral load in oropharyngeal samples as assessed by RT-PCR in samples collected at baseline, 48 and 96 hours after randomization

Secondary endpoints:

- Change in National Early Warning Score (NEWS) from randomization to 96 hours
- Admission to ICU
- In-hospital mortality
- Duration of hospital admission
- Mortality at 30 and 90 days
- Clinical status as assessed by percentage of subjects reporting each severity rating on a 7-point ordinal scale on day 14 after study inclusion:

1. Death
2. Hospitalized, on invasive mechanical ventilation or extracorporeal membrane oxygenation
3. Hospitalized, on non-invasive ventilation or high flow oxygen devices
4. Hospitalized, requiring supplemental oxygen
5. Hospitalized, not requiring supplemental oxygen
6. Not hospitalized, but unable to resume normal activities
7. Not hospitalized, with resumption of normal activities

- Change in protein biomarker profiles from randomization to 96 hours, including C-reactive protein, markers of renal and hepatic injury, and established cardiac biomarkers like cardiac troponin and B-type natriuretic peptide concentrations

Study Design:

Two-arm, open label, single center, pragmatic study with possible extension to other Norwegian sites, pending regulatory approvals.

Main Inclusion Criteria:

1. Hospitalised
2. Adults 18 year or older
3. Moderately severe disease (NEWS score ≤ 6)
4. SARS-CoV-2 positive nasopharyngeal swab
5. Expected time of admission > 48 hours
6. Signed informed consent must be obtained and documented according to ICH GCP, and national/local regulations.

Main Exclusion Criteria	1. Requiring ICU admission at screening<br>2. History of psoriasis<br>3. Known adverse reaction to hydroxychloroquine sulphate<br>4. Pregnancy<br>5. Prolonged QT interval (>450 ms)
Sample Size:	Pilot inclusion of 51 patients, with subsequent inclusion and analyses after 101, 151 and 202 completed patients.
Efficacy Assessments:	Viral kinetics 96 hours after randomization.
Safety Assessments:	Assessment of Adverse Events (AE), Serious Adverse Events (SAE) and Suspected Unexpected Serious Adverse Reactions (SUSAR).

TABLE OF CONTENTS

CONTACT DETAILS 2
SIGNATURE PAGE 4
SIGNATURE PAGE SITE INVESTIGATORS 5
PROTOCOL SYNOPSIS 6
TABLE OF CONTENTS 9
LIST OF ABBREVIATIONS AND DEFINITIONS OF TERMS 12
1 INTRODUCTION 12
1.1 Background – Disease 12
1.2 Background - Therapeutic Information 12
1.3 Pre-Clinical & Clinical Experience with Investigational Medicinal Product (IMP) 13
1.4 Rationale for the Study and Purpose 13
2 STUDY OBJECTIVES AND RELATED ENDPOINTS 13
Primary Endpoint 14
Secondary Endpoints 14
3 OVERALL STUDY DESIGN 14
4 STUDY POPULATION 16
4.1 Selection of Study Population 16
4.2 Number of Patients 16
4.3 Inclusion Criteria 16
4.4 Exclusion Criteria 16
5 TREATMENT 16
5.1 Drug Identity, Supply and Storage 17
5.2 Dosage and Drug Administration 17
5.3 Duration of Therapy 17
5.4 Concomitant Medication 17
5.5 Subject Compliance 17
5.6 Drug Accountability 17
5.7 Drug Labelling 17
5.8 Subject Numbering 17
6 STUDY PROCEDURES 18
6.1 Flow Chart 18
6.2 Criteria for Patient Discontinuation 18
6.3 Procedures for Discontinuation 19
6.3.1 Patient Discontinuation 19
6.3.2 Trial Discontinuation 19
6.4 Laboratory Tests 19

6.5 Clinical variables 19
7 ASSESSMENTS 21
7.1 Assessment of Efficacy / Response 21
7.2 Safety and Tolerability Assessments 21
8 SAFETY MONITORING AND REPORTING 22
8.1 Definitions 22
8.1.1 Adverse Event (AE) and Adverse Events of Special Intrest (AESI) 22
8.1.2 Serious Adverse Event (SAE) 22
8.1.3 Suspected Unexpected Serious Adverse Reactions (SUSAR) 22
8.2 Expected Adverse Events 23
8.3 Time Period for Reporting AESI and SAE 23
8.4 Recording of Serious AEand AESI 23
8.5 Reporting Procedure 24
8.5.1 SAEs and AESIs 24
8.5.2 SUSARs 24
8.6 Risk–benefit 24
8.7 Data Monitoring Committee (DMC) 24
9 DATA MANAGEMENT AND MONITORING 25
9.1 Electronic Case Report Forms (eCRF) 25
9.2 Source Data 25
9.3 Study Monitoring 26
9.4 Confidentiality 26
9.5 Database management 26
10 STATISTICAL METHODS AND DATA ANALYSIS 27
10.1 Determination of Sample Size 27
10.2 Randomization 28
10.2.1 Allocation- sequence generation 28
10.2.2 Allocation- procedure to randomize a patient 28
10.2.3 Blinding and emergency unblinding 28
10.3 Population for Analysis 28
10.4 Planned analyses 28
10.5 Statistical Analysis 29
10.5.1 Primary analysis 29
10.5.2 Secondary analyses 29
10.5.3 Safety analyses 29
10.5.4 Descriptive statistics 29
10.5.5 Missing data 30
11 STUDY MANAGEMENT 30

11.1 Investigator Delegation Procedure 30
11.2 Protocol Adherence 30
11.3 Study Amendments 30
11.4 Audit and Inspections 30
12 ETHICAL AND REGULATORY REQUIREMENTS 30
12.1 Ethics Committee Approval 30
12.2 Other Regulatory Approvals 30
12.3 Informed Consent Procedure 31
12.4 Subject Identification 31
13 TRIAL SPONSORSHIP AND FINANCING 31
14 TRIAL INSURANCE 31
15 PUBLICATION POLICY 31
16 REFERENCES 32
17 LIST OF APPENDICES 32

LIST OF ABBREVIATIONS AND DEFINITIONS OF TERMS

Abbreviation or special term	Explanation
AE	Adverse Event
CRF	Case Report Form (electronic/paper)
CSA	Clinical Study Agreement
DAE	Discontinuation due to Adverse Event
EC	Ethics Committee, synonymous to Institutional Review Board (IRB) and Independent Ethics Committee (IEC)
GCP	Good Clinical Practice
ICF	Informed Consent Form
ICH	International Council for Harmonization
IMP	Investigational Medicinal Product (includes active comparator and placebo)
ITT	Intention to treat
PP	Per-protocol
SAE	Serious Adverse Event
SAP	Statistical Analysis Plan
SOP	Standard Operating Procedure

1 INTRODUCTION

1.1 Background – Disease

On 30 December 2019, bronchoalveolar lavage samples were collected from a patient with pneumonia of unknown ethology in Wuhan, China. Real-time polymerase chain reaction (RT-PCR) assays on these samples were positive for pan-Betacoronavirus and genome sequencing indicated that the virus had the closest relationship with the bat SARS-like coronavirus strain BatCov RaTG1. The new virus was named severe acute respiratory syndrome coronavirus 2 (SARS-CoV-2) and the respiratory disease it causes COVID-19 *(1)*.

COVID-19 is transmitted via droplets and fomites during close unprotected contact between an infector and infectee and has per March 2020 been detected in approximately 126 000 people worldwide with most cases appearing in the Hubei province of China, South Korea, Iran and Italy. On March 11 2020, the WHO declared COVID-19 a global pandemic *(2)*. In Norway, 629 cases have been detected, and the first patients have been admitted to hospitals and ICUs. Approximately 80% of laboratory confirmed patients in China have had mild to moderate disease, 13.8% have had severe disease and 6.1% critical. The case fatality rate in China has been 3.8 % (5.8% in the city of Wuhan and 0.7% in the remaining China). Mortality increases with age, is higher in men than women and higher in those with important comorbidity (cardiac disease, diabetes mellitus, chronic respiratory disease, cancer and hypertension).

1.2 Background - Therapeutic Information

To date, there is no specific therapy for COVID-19. However, supportive treatment including oxygen supplementation and mechanical intervention is believed to strongly effect the course of the disease. The effect of several antivirals are

currently being investigated in clinical including remdesivir, lopinavir/ritonavir (HIV protease inhibitor), rintatolimod (immune modulator), danoprevir+ritonavir (HIV protease inhibitor), Galidesivir (broad spectrum antiviral drug) and remdesivir (a nucleoside inhibitor of coronavirus polymerase) *(3)*. In addition, chloroquine has been suggested as a treatment against COVID-19.

1.3 Pre-Clinical & Clinical Experience with Investigational Medicinal Product (IMP)

Chloroquine is an important anti-malaria drug that has been used since 1947. Furthermore, since the late seventies it has been used as treatment against rheumatoid arthritis and other rheumatic disorders. In short term use, chloroquine has a good safety profile. Its most important side effect being reversible accommodation disturbances and swelling of the cornea. In long term use (months) it may cause chronically reduced vision.

The effect of chloroquine on SARS-CoV-2 is supported by *in vitro* data showing a strong antiviral effect in non-toxic doses in cell culture *(4)* and by efficacy in treatment of COVID-19 associated pneumonia in clinical studies *(5)*. The antiviral mechanism of chloroquine is associated with increased endosomal pH needed for fusion-mediated viral entry into host cells, and with alteration of glycosylation of SARS-CoV-2 cellular receptors. Accordingly, the Chinese Expert Consensus on Chloroquine Phosphate for the Treatment of Novel Coronavirus Pneumonia recently recommended short term treatment with chloroquine in patients with mild, moderate and severe cases of novel coronavirus pneumonia and without contraindications to chloroquine use *(6)*.

1.4 Rationale for the Study and Purpose

We hypothesize that early treatment with chloroquine in patients with established COVID-19 is safe and will significantly increase the virological clearance rate of SARS-CoV-2. Furthermore, we hypothesize that early treatment with chloroquine is associated with more rapid resolve of clinical symptoms and a decreased admission rate to intensive care units and in-hospital mortality. Considering the immediate and worldwide health emergency associated with the SARS-CoV-2 outbreak and the current lack of evidence based medical interventions for this patient group, studies investigating possible treatment modalities in COVID-19 are direly needed.

2 STUDY OBJECTIVES AND RELATED ENDPOINTS

Specific patient groups, especially elderly patients with comorbidities, are at higher risk of contracting viral respiratory disease and hospitalization than the general population. Relevant clinical end points depend on the patient populations studied, local standards of care, and physician judgment. Studies that focus solely on ICU admission or mortality as primary outcomes are likely to require prolonged time frames for accrual of sufficient numbers of patients and also need to consider many confounding variables that affect clinical outcomes in a possibly heterogeneous patient group. Measures of rate of decline of virus replication as primary end points to evaluate and compare drug efficacy of antivirals is logical and necessary, especially in heterogeneous populations such as hospitalized and immunocompromised patients. Rapid reductions in active viral replication may be essential to prevent tissue damage and to further clinical recovery, as well as reduce risk of viral complications and mortality *(7)*.

The recent years National Early Warning Score (NEWS) has been implemented in Norwegian hospitals as a tool to assess the degree of illness in a patient and to guide level of surveillance and intensity of treatment. This method is based on the degree of abnormality of the vital signs respiratory rate, oxygen saturation, body temperature, systolic blood pressure and heart rate, in addition to the need for supplemental oxygen and mental alertness *(8)*. Scores range from 0-20, with a higher score representing further removal from normal physiology and a higher risk of morbidity and mortality. We expect that approximately 75% of patients will be discharged by day 14 *(9)* and will therefore analyse clinical status at this day.

Accordingly, the **primary aim** of this study is to assess whether treatment with chloroquine in patients with COVID-19 will increase the decline rate of SARS-CoV-2 in the oropharynx from baseline to 48 and 96 hours. As described above, the use of primary virological end points is strongly supported by the current literature on novel antiviral therapy.

The **secondary aims** are to assess whether treatment with chloroquine in patients with COVID-19 (1) will improve clinical status as assessed by the NEWS score, improve clinical outcomes, including (2) admission rate to intensive care units and (3) in-hospital mortality, and (4) improve protein biomarker profile (inflammation, cardiac, renal, hepatic).

Specific objectives

- Assess the impact of early treatment with chloroquine in patients with established COVID-19 on SARS-CoV-2 presence in oropharyngeal specimens at 48 and 96 hours after inclusion in the trial
- Assess the impact of early treatment with chloroquine in patients with established COVID-19 on changes in NEWS score
- Assess the impact of early treatment with chloroquine in patients with established COVID-19 on admission rate to ICU
- Assess the impact of early treatment with chloroquine in patients with established COVID-19 on in-hospital mortality and mortality after 30 and 90 days
- Assess the impact of early treatment with chloroquine in patients with established COVID-19 on clinical outcomes after 14 days
- Assess the impact of early treatment with chloroquine in patients with established COVID-19 on markers of inflammation, and cardiac, renal and hepatic injury at 96 hours after inclusion in the trial

Primary Endpoint

- Rate of decline in SARS-CoV-2 viral load in oropharyngeal samples as assessed by RT-PCR in samples collected at baseline, 48 and 96 hours after randomization

Secondary Endpoints

- Change in NEWS score at 96 hours after randomization
- Admission to ICU
- In-hospital mortality
- Duration of hospital admission
- Mortality at 30 and 90 days
- Clinical status will be assessed by percentage of subjects reporting each severity rating on a 6-point ordinal scale 14 days after randomization:
  1. Death
  2. Hospitalized, on invasive mechanical ventilation or extracorporeal membrane oxygenation
  3. Hospitalized, on non-invasive ventilation or high flow oxygen devices
  4. Hospitalized, requiring supplemental oxygen
  5. Hospitalized, not requiring supplemental oxygen
  6. Not hospitalized, but unable to resume normal activities
  7. Not hospitalized, with resumption of normal activities
- Change in protein biomarker profiles at 96 hours after randomization, including C-reactive protein, markers of renal and hepatic injury, and established cardiac biomarkers like cardiac troponin and B-type natriuretic peptide concentrations

3 OVERALL STUDY DESIGN

The study is a two-arm, open label, pragmatic randomized controlled trial (RCT) designed to assess the virological and clinical effect of chloroquine therapy in addition to standard of care versus standard of care in patients with established

COVID-19. Pragmatic clinical trials (PCT) are characterized by 3 attributes: (1) focus on informing decision-makers (e.g. patients, politicians, administrators) on optimal clinical medicine practice, as opposed to elucidating a biological or social process; (2) intent to enrol a population representative to the decision in practice and for whom the decision is relevant; and (3) either an intent to streamline procedures and data collection in the trial or to measure a broad range of outcomes. By utilizing resources already paid for by the hospitals (physicians and nurses in daily clinical practice), pragmatic clinical trials can include a larger number of patients at a short time duration and at a lower cost than studies utilizing traditional RCT designs with an external study organization (e.g. study nurses, study physicians). Due to the immediate need for study commencement and the time frame of the current proposal, a pragmatic approach will enable swift initiation of randomization and treatment. We will especially use data from the data warehouse at Akershus University Hospital for eligible patient identification (i.e. *electronic surveillance*) and for automatic data extraction to the study specific database. The study will not be able to procure an acceptable placebo treatment and the study will accordingly not be placebo-controlled.

In the initial phase of the study, patients will be included from a single center (Akershus University Hospital). The study is designed as a sequential adaptive trial where interim efficacy analyses are planned after the inclusion of 51 patients, with subsequent analyses after 101, 151 and 202 completed patients. This approach will enable frequent assessment of all outcome measures. After inclusion of the initial 51 patients, analyses will be performed for the predefined outcome measures. Pending these results, the study will possibly be extended to other Norwegian centres, pending separate submission to ethics committee and other regulatory bodies from new centers. See Section 10 Statistical methods and data analysis for further details.

All patients at Akershus University Hospital with suspicion of acute respiratory tract infections are examined with a nasopharyngeal swab, with subsequent microbiological examination, including SARS-CoV-2 specific polymerase chain reaction (PCR). Laboratory reports from the Department of Microbiology are surveilled real-time for SARS-CoV-2 positive samples in the local data warehouse, which will allow for immediate screening and randomization in all eligible subjects. **Figure 1** gives a schematic overview of the study.

Figure 1. Trial design

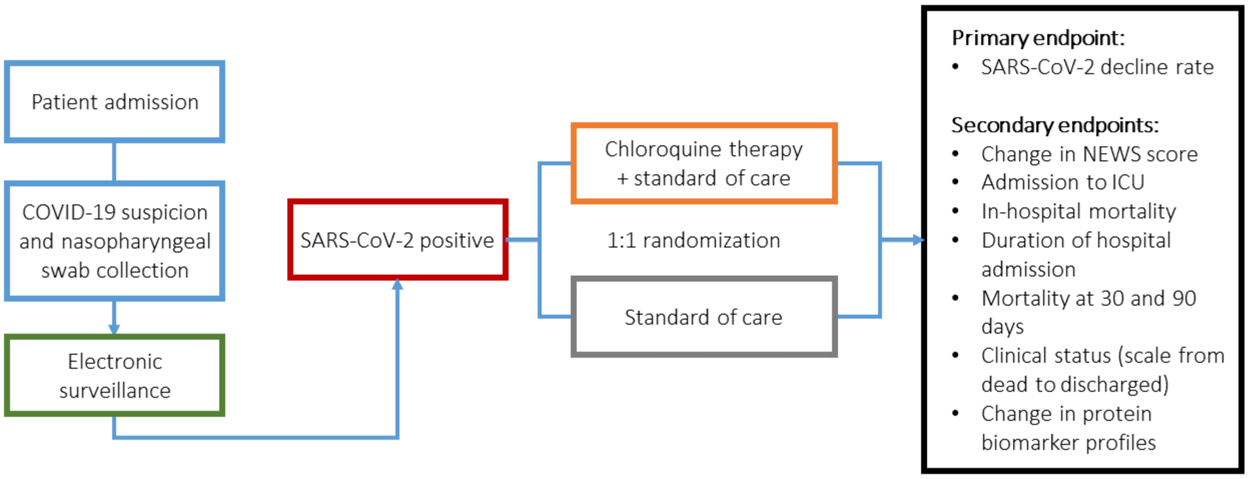

Study Period of initial study phase:	Estimated date of first patient enrolled: 23.03.20<br>Anticipated recruitment period: 6 months<br>Estimated date of last patient completed: 01.04.21<br>Estimated study termination: 23.03.25
Treatment Duration:	Patients allocated to the treatment arm will be treated with 400 mg hydroxychloroquine sulphate (equalling 310 mg base) twice daily for seven days
Follow-up:	Continuous of follow-up at admission, as well as assessment of clinical status at 14 days and mortality at day 30 and 90

4 STUDY POPULATION

4.1 Selection of Study Population

Participants will be recruited from the entirety of the inpatients at the participating hospitals. Electronic real-time surveillance of laboratory reports from the Department of Microbiology will be examined regularly, with maximum interval 24 hours, for SARS-CoV-2 positive subjects.

4.2 Number of Patients

We aim to include patients by a sequential adaptive approach, where analyses are planned after the inclusion of 51 patients, with subsequent interim efficacy analyses after 101, 151 and 202 completed patients. All patients included in each sequence will be used for the final analyses of the entire study. See Section 10 Statistical methods and data analysis for further details.

4.3 Inclusion Criteria

All of the following conditions must apply to the prospective patient at screening prior to inclusion:

1. Hospitalised
2. Adults 18 year or older
3. Moderately severe disease (NEWS score ≤ 6)
4. SARS-CoV-2 positive nasopharyngeal swab
5. Expected time of admission > 48 hours
6. Signed informed consent must be obtained and documented according to ICH GCP, and national/local regulations.

4.4 Exclusion Criteria

Patients will be excluded from participation in the study if they meet any of the following criteria:

1. Requiring ICU admission at screening
2. History of psoriasis
3. Known adverse reaction to hydroxychloroquine sulphate
4. Pregnancy
5. Prolonged QT interval (>450 ms)

5 TREATMENT

Recent reports document inhibition of SARS-CoV-2 by chloroquine *in vitro*, as well as in experimental clinical trials conducted in China. No apparent adverse effects of chloroquine treatment has been observed in these trials on human subjects. Hydroxychloroquine has a more favourable side effect profile compared to chloroquine and is more potent than chloroquine to inhibit SARS-CoV-2 *in vitro (10)*. Accordingly, in the current proposal, and in accordance with the Chinese Expert Consensus on Chloroquine Phosphate for the Treatment of Novel Coronavirus Pneumonia *(6)*, we will utilize a short term treatment with 400 mg hydroxychloroquine sulphate (equalling 310 mg base) twice daily for seven days.

Standard of care will apply to both treatment arms at the discretion of the treating physician. Due to the lack of randomized evidence based therapy in COVID-19 and possibly a large proportion of patients who will present with serious to life-threatening disease, treatment by compassion will be allowed at any time at the discretion of the treating physician.

5.1 Drug Identity, Supply and Storage

For this study, hydroxychloroquine sulphate is defined as the Investigational Medicinal Product (IMP). The IMP has marketing authorization as a medicinal product, is produced by *Sanofi Aventis* under the trade name “Plaquenil”, and is distributed through licensed pharmacists.

The drug will be provided by the trial sponsor (Akershus University Hospital) to patients included in the treatment arm free of charge.

5.2 Dosage and Drug Administration

At enrolment, all patients randomized to treatment will be allocated a per oral dosage of 400 mg hydroxychloroquine sulphate (equalling 310 mg base) twice daily for seven days. This is in agreement with the SPC of the IMP. The drug will be dispensed at the regular drug administration rounds of the clinical wards.

5.3 Duration of Therapy

Duration of therapy is per protocol seven days, equalling 14 doses of 400 mg hydroxychloroquine sulphate (equalling 310 mg base). The duration of treatment is based on a Chinese Expert Consensus that 5-10 days of chloroquine should be administered to patients with coronavirus pneumonia *(6)*.

5.4 Concomitant Medication

All concomitant medication used by the patient will be recorded at admission and retrieved from the hospital electronic prescribing system. Care should be taken when patients are on antiepileptic or antidiabetic medication, as well as on therapy associated with risk of QT time prolongation.

5.5 Subject Compliance

Compliance to the protocol will be monitored by the hospital electronic prescribing system, where administration status of every dose prescribed is registered.

5.6 Drug Accountability

The IMP has a marketing authorization in Norway, is routinely ordered by the pharmacy and will be dispensed from the pharmacy's own stock. Dosage, batch number and shelf life will be noted from all packages of the dispensed IMPs.

5.7 Drug Labelling

The IMP is labeled according to the standard production by the commercial manufacturer (*Sanofi Aventis).*

5.8 Subject Numbering

Each subject is identified in the study by a unique subject number that is assigned when subject signs the Informed Consent Form. Once assigned the subject number cannot be reused for any other subject.

6 STUDY PROCEDURES

As described in Section 3, the current study will utilize a pragmatic approach. In this regard, we will utilize resources already paid for by the hospitals, and all study procedures will be performed at the discretion of the treating physician, including laboratory testing, medical imaging and other procedures deemed clinically necessary.

6.1 Flow Chart

Table. Trial flow chart

			**Time after randomization**											
	**Screening**	**Randomization**	**24 h**	**48 h**	**72 h**	**96 h**	**120 h**	**144 h**	**168 h**	**192 h**	**216 h**	**14 d**	**30 d**	**90 d**
Pharyngeal swab	X	[1]X		[2]X		[2]X		[2]X						
Inclusion/exclusion evaluation		X												
Informed consent		X												
Study treatment		X	X	X	X	X	X	X	X					
Physical examination	[3]X		[4]X	[4]X	[4]X	[4]X	[4]X	[4]X	[4]X	[4]X	[4]X			
Blood samples	[4, 5]X	[4]X	[4]X	[4]X	[4]X	[4]X	[4]X	[4]X	[4]X	[4]X	[4]X			
AESI and SAEs[6-8]			X	X	X	X	X	X						
ECG	X		[9]X	[9]X	[9]X	[9]X	[9]X	[9]X	[9]X	[9]X	[9]X			
Record of medication[10]		X												
Mortality			X	X	X	X	X	X	X	X	X		X	X
Phone follow-up								[11]X						
Clinical status												[12]X		

[1]If > 12 hours since screening nasopharyngeal swab
[2]Study specific biobanking
[3]Clinical examination at the discretion of the admitting physician (full clinical examination including heart, lungs, abdomen, extremities), as well as medical history, substance use, allergies, current medication, etc.
[4]At the discretion of the treating physician
[5]Women of child bearing age will be tested by serum hCG for possible pregnancy
[6]Every day whilst under treatment
[7]AESIs for this protocol is defined as gastrointestinal discomfort, visual disturbances, diarrhoea, headache, nausea and dizziness
[8]If discharged before end of treatment, assessment of AESI and SAE will be perfomed by phone directly to the patient
[9]Daily ECG as long as a drug known to prolong QT interval is taken in combination with chloroquine
[10]Current medication at admission and antimicrobial therapy initiated at admission
[11]If discharged before 96 hours
[12]Death, hospitalized (on invasive mechanical ventilation or extracorporeal membrane oxygenation), hospitalized (on non-invasive ventilation or high flow oxygen devices), hospitalized (requiring supplemental oxygen), hospitalized (not requiring supplemental oxygen), not hospitalized

6.2 Criteria for Patient Discontinuation

Patients may be discontinued from the study any time. Specific reasons for discontinuing a patient for this study are:

- Voluntary discontinuation by the patient who is at any time free to discontinue his/her participation in the study, without prejudice to further treatment.
- Incorrect enrolment i.e. the patient does not meet the required inclusion/exclusion criteria for the study
- In the occurrence of severe adverse events, possibly or probably associated to the IMP, the IMP should be stopped unless a clinical risk/benefit assessment at the treating physician's discretion warrants continuation.

6.3 Procedures for Discontinuation

6.3.1 Patient Discontinuation

The reason for discontinuation will be recorded, if the patient chooses to disclose it. Management of patients who withdraw or are withdrawn from the study will have to be individualized and should in all cases be discussed with the Principal Investigator. All patients randomized will be included in the study population. The following may trigger individual patient discontinuation from the trial:

- When chloroquine is used in combination with drug therapy known to prolong QT interval, an increase in QTc above 470ms for males or 480ms for females, or the QTc interval increases 60 ms or more from pre-treatment values *(11)*.

6.3.2 Trial Discontinuation

The whole trial may be discontinued at the discretion of the sponsor in the event of any of the following:

- Occurrence of AEs unknown to date in respect of their nature, severity and duration that may negatively affect the benefit/risk of the trial.
- Medical or ethical reasons affecting the continued performance of the trial
- Difficulties in the recruitment of patients

The sponsor and principal investigator(s) will inform all investigators, the relevant Competent Authorities and Ethics Committees of the termination of the trial along with the reasons for such action. If the study is terminated early on grounds of safety, the Competent Authorities and Ethics Committees will be informed within 15 days.

6.4 Laboratory Tests

As described in Section 3, the current study will utilize a pragmatic approach. In this regard, we will utilize resources already paid for by the hospitals, among others laboratory tests, except for quantitative virological investigations and a pregnancy test of women of childbearing age. All laboratory sampling will be done at the discretion of the treating physician. Glucose concentrations are routinely measured in all admitted patient with diabetes mellitus and/or on antidiabetic therapy. On admission, a standard panel of laboratory test will be performed on all patients (covering haematology, electrolytes, liver- and renal function, standard serology, inflammatory markers), and these will be performed locally in accordance with hospital/laboratory standard procedures. The samples will be labelled with study specific ID only (i.e. the samples will be handled de-identified).

A specific research biobank will be established in accordance with national regulations. Oropharyngeal swabs will be collected in a uniform fashion at randomization if > 12 hours have passed since the screening sample, as well as at 48 hours and 96 hours after randomization. Nasopharyngeal swab at screening from the clinical laboratories will additionally be transferred to the research biobank upon study inclusion, and stored in an ultra-freezer at Akershus University Hospital.

6.5 Clinical variables

Collection of clinical variables will start at admission and for the entirety of the hospital stay. Data will be collected from the hospital electronic record system, including electronic patient records, laboratory and medical imaging systems, and prescribing systems. The data warehouse at Akershus University Hospital will be utilized for automatic data extraction to the study specific database. All clinical variables will be registered in the study eCRF system, including clinical endpoints and quantitative virological results from serial oropharyngeal specimens.

Data retrieved from hospital electronic record system

Date of birth	
Sex	
Admission date	
Discharge date	
Date of symptom onset	
Admission signs and symptoms	Fever<br>Chills<br>Cough<br>Sputum production<br>Sore throat<br>Chest pain<br>Shortness of breath<br>Headache<br>Confusion<br>Myalgia or arthralgia<br>Abdominal pain<br>Diarrhoea<br>Vomiting/Nausea<br>Skin rash
Epidemiological factors	History of travel to an area with documented cases of COVID-19 infection?<br>Contact with a confirmed/probable case of a symptomatic COVID-19 patient?
Imaging on admission (chest X-ray, CT, etc)	Infiltrates<br>Pleural effusion<br>Ground glass opacities
Co-morbidities	Cardiovascular disease<br>Chronic obstructive pulmonary disease<br>Chronic kidney disease<br>Autoimmune disease<br>Diabetes mellitus<br>Cognitive impairment/dementia<br>Cancer
Substance use	Tobacco and alcohol consumption
Clinical biochemistry (upon availability, on admission and as long as admitted)	Haemoglobin<br>Platelet count<br>Leukocyte count with differential count<br>C-reactive protein (CRP)<br>Glucose<br>Na+<br>K+<br>Creatinine with estimated glomerular filtration rate<br>Urea<br>International Normalized Ratio (INR)<br>Bilirubin<br>Alanine aminotransferase (ALT)<br>Aspartate aminotransferase (AST)<br>Alkaline phosphatase (ALP)<br>Gamma-glutamyl transferase (GT)<br>NT-proBNP<br>Troponin T<br>D-dimer<br>APTT<br>Fibrinogen<br>Procalcitonin<br>Albumin

	LD<br>pH (from arterial blood gas)<br>pCO2 (from arterial blood gas)<br>pO2 (from arterial blood gas)<br>sO2 (from arterial blood gas)<br>Bicarbonate (HCO3) (from arterial blood gas)<br>Base excess (from arterial blood gas)<br>Lactate (from arterial blood gas)
Results from microbiology	Nasofaryngeal specimen (specific vira apart from SARS-CoV-2)<br>Blood culture results
Anthropometrics	Height (cm)<br>Weight (kg)
ECG recordings (upon availability, on admission and as long as admitted)	Heart rate, rhythm, intervals, ST-T assessment, pathology
Echocardiography (upon availability, on admission and as long as admitted)	Left and right ventricular structure and function, pericardial effusion, pulmonary pressure, other pathology
Daily NEWS score on three separate time point with > 5 hours apart (at regular nurse rounds)	Respiratory rate<br>Oxygen saturation<br>Supplemental oxygen<br>Temperature<br>Systolic blood pressure<br>Heart rate<br>Level of consciousness (alert, verbal, pain, unresponsive)
Medical therapy/medication	Current medication at admission<br>Antimicrobial therapy initiated at admission
Clinical outcomes	Admission to ICU<br>Mortality (in-hospital, after 30 and 90 days)<br>Hospitalization status 14 days after randomization<br>1. Death<br>2. Hospitalized, on invasive mechanical ventilation or extracorporeal membrane oxygenation<br>3. Hospitalized, on non-invasive ventilation or high flow oxygen devices<br>4. Hospitalized, requiring supplemental oxygen<br>5. Hospitalized, not requiring supplemental oxygen<br>6. Not hospitalized, but unable to resume normal activities<br>7. Not hospitalized, with resumption of normal activities
Safety monitoring and reporting	Adverse events<br>Serious adverse events<br>Suspected unexpected serious adverse reactions

7 ASSESSMENTS

7.1 Assessment of Efficacy / Response

Viral kinetics at 96 hours after randomization is the primary efficacy measure. We will assess the kinetics of SARS-CoV-2 by serial oropharyngeal samples with quantification of viral load, measured by RT-PCR ($\log_{10}$ copies/mL) *(12)*.

7.2 Safety and Tolerability Assessments

Safety will be monitored by the assessments described below as well as the collection of AEs, SAEs and SUSARs. For details on collection and reporting, refer to Section 8.

8 SAFETY MONITORING AND REPORTING

The investigator is responsible for the detection and documentation of events meeting the criteria and definition of an adverse event (AE) or serious adverse event (SAE). The methods for collection of safety data are described below.

8.1 Definitions

8.1.1 Adverse Event (AE) and Adverse Events of Special Interest (AESI)

An adverse event (AE) is any untoward medical occurrence in a patient in relation to administration of a pharmaceutical product and which does not necessarily have a causal relationship with this treatment.

An AE can therefore be any unfavourable and unintended sign (including an abnormal laboratory finding), symptom, or disease temporally associated with the use of a medicinal (investigational) product, whether or not related to the medicinal (investigational) product.

The term AE is used to include both serious and non-serious AEs.

If an abnormal laboratory value/vital sign are associated with clinical signs and symptoms, the sign/symptom should be reported as an AE.

AESIs for this protocol is defines as gastrointestinal discomfort, visual disturbances, diarrhoea, headache, nausea and dizziness.

8.1.2 Serious Adverse Event (SAE)

Any untoward medical occurrence that at any dose:

- Results in death
- Is immediately life-threatening
- Requires in-patient hospitalization or prolongation of existing hospitalization
- Results in persistent or significant disability or incapacity
- Is a congenital abnormality or birth defect
- Is an important medical event that may jeopardize the subject or may require medical intervention to prevent one of the outcomes listed above,

Medical and scientific judgment is to be exercised in deciding on the seriousness of a case. Important medical events may not be immediately life-threatening or result in death or hospitalization, but may jeopardize the subject or may require intervention to prevent one of the listed outcomes in the definitions above. In such situations, or in doubtful cases, the case should be considered as serious.

8.1.3 Suspected Unexpected Serious Adverse Reactions (SUSAR)

Suspected Unexpected Serious Adverse Reaction: SAE (see section 8.1.2) that is unexpected as defined in section 8.2 and possibly related to the IMP. Any event other than those mentioned in the SPC of the IMP may be classified as SUSAR.

8.2 Expected Adverse Events

The most common SAE in short-term therapy with the IMP is gastrointestinal discomfort. In addition, the following symptoms will be registered in the CRF: visual disturbances, diarrhoea, headache, nausea and dizziness. These AE and gastrointestinal discomfort is considered adverse events of special interest (AESI) and will be reported according to standard adverse event criteria. On day 1, 3 and 6 during the treatment patients will be asked specifically about AESI. Further adverse events listed in the SPC for the IMP will also be registered as AE/SAE if they occur (see Appendix Summary of Product Characteristics “PLAQUENIL”).

8.3 Time Period for Reporting AESI and SAE

For each patient the standard time period for collecting and recording SAEs will begin at the day of IMP treatment start and will continue for the entirety of the duration of treatment. In the case of patient discharge before the end of treatment, patients will be followed up by phone for assessment of AEs. During the course of the study all SAEs will be proactively followed up for each patient; events should be followed up to resolution, unless the event is considered by the investigator to be unlikely to resolve due to the underlying disease. Every effort should be made to obtain a resolution for all events.

Except AESI, AEs will not be reported, unless they meet the definition of an SAE. Events recognized by the treating physician as a consequence of the natural clinical course of the disease will not be treated as an SAE (e.g. respiratory deterioration, hypotension, myalgia).

8.4 Recording of Serious AE and AESI

If the patient has experienced serious adverse event(s), the investigator will record the following information in the CRF:

- The nature of the event(s) will be described by the investigator in precise standard medical terminology and assigned an ICD-10 diagnosis (i.e. not necessarily the exact words used by the patient).
- The duration of the event will be described in terms of event onset date and event ended data.
- The intensity of the adverse event will be graded as follows (18):
  - Mild: asymptomatic or mild symptoms; clinical or diagnostic observations only; intervention not indicated.
  - Moderate: minimal, local or non-invasive intervention indicated; limiting age-appropriate instrumental Activities of Daily Living (ADL)
  - Severe or medically significant but not immediately life-threatening: hospitalization or prolongation of hospitalization indicated; disabling; limiting self-care ADL.
- The causal relationship of the event to treatment will be assessed as one of the following:

  Unrelated:
  There is not a temporal relationship to treatment, or there is a reasonable causal relationship between non-investigational product, concurrent disease or circumstance and the AE.

  Unlikely:
  There is a temporal relationship to treatment, but there is not a reasonable causal relationship between treatment and the AE.

  Possible:
  There is reasonable causal relationship between treatment and the AE.

  Probable:
  There is a reasonable causal relationship between treatment and the AE.

Definite:
There is a reasonable causal relationship between treatment and the AE.

- Action taken.
- The outcome of the adverse event – whether the event is resolved or still ongoing.

It is important to distinguish between serious and severe AEs. Severity is a measure of intensity whereas seriousness is defined by the criteria in Section 8.1. An AE of severe intensity need not necessarily be considered serious. For example, nausea that persists for several hours may be considered severe nausea, but is not an SAE. On the other hand, a stroke that results in only a limited degree of disability may be considered a mild stroke, but would be an SAE. AEs with a reasonable causal relationship to the treatment will be reported in a separate AE form.

8.5 Reporting Procedure

8.5.1 SAEs and AESIs

All adverse events of special interest and serious adverse events that should be reported as defined in section 8.1.1 and 8.1.2 will be recorded in the patient's CRF.

SAEs must be reported within 24 hours after the site has gained knowledge of the SAE. Every SAE must be documented by the investigator on the AE forms in Viedoc and signed electronically. The initial report shall promptly be followed by detailed, written reports if necessary. The initial and follow-up reports shall identify the trial subjects by unique code numbers assigned to the latter.

The coordinating investigator keeps detailed records of all SAEs reported by the investigators and performs an evaluation with respect to causality and expectedness. Based on, among other, SAE reports the sponsor will evaluate whether the risk/benefit ratio associated with study is changed.

8.5.2 SUSARs

SUSARs will be reported to the Norwegian Medicines Agency and REK Sør-Øst according to national regulation. The sponsor will ensure that all relevant information about SUSARs that are fatal or life-threatening is recorded and reported as soon as possible and in no case later than seven (7) days after knowledge by the sponsor of such a case, and that relevant follow-up information is subsequently communicated within an additional eight (8) days.

SUSARs will be reported to The Norwegian Medicines Agency (post@legemiddelverket.no) using the CIOMS form no later than 7 days after the incident.

8.6 Risk–benefit

As described previously, the inherent risk associated with IMP is low, and expected adverse events are mild and self-limiting especially in short-term use. The risk of mortality from COVID-19 is yet undetermined, but preliminary reports document significant mortality risk, especially in elderly with pre-existing chronic disease. WHO has declared COVID-19 a global pandemic, and novel measures aimed at decreasing morbidity and mortality are imminently needed. Accordingly, the possible benefit of the IMP on individual, national and international scale greatly outweighs individual risk.

8.7 Data Monitoring Committee (DMC)

A data monitoring committee (DMC) will be established to monitor the safety and efficacy of the study treatment and will consist of one statistician and two physicians with experience from virology/infectious disease and clinical studies. All members will be independent from the sponsor and will not be investigators or collaborators in the current study. The

DMC will review all SAEs. The study investigators may call on the DMC to review specific SAEs. The DMC will conduct its tasks according to the EMA guideline (http://www.ema.europa.eu/docs/en_GB/document_library/Scientific_guideline/2009/09/WC500003635.pdf). A DMC charter based on the NORCRIN (http://www.norcrin.no/dokumentoversikt/) template will be signed and included in the Trial Master File.

9 DATA MANAGEMENT AND MONITORING

9.1 Electronic Case Report Forms (eCRF)

The Clinical Data Management System (CDMS) used for the eCRF in this study is Viedoc. The eCRF system will be FDA Code of Federal Regulations 21 Part 11 compliant.

The designated investigator staff will enter the data required by the protocol into the eCase report forms (eCRF). The Investigator is responsible for assuring that data entered into the eCRF is complete, accurate, and that entry is performed in a timely manner. The signature of the investigator will attest the accuracy of the data on each eCRF. If any assessments are omitted, the reason for such omissions will be noted on the eCRFs. Corrections, with the reason for the corrections will also be recorded.

After database lock, the investigator will receive a digital copy of the subject data for archiving at the investigational site.

9.2 Source Data

Source data are all information in original records and certified copies of original records of clinical findings, observations, or other activities in a clinical trial necessary for the reconstruction and evaluation of the trial. Source data are contained in source documents (original records or certified copies).

The medical records for each patient should contain information which is important for the patient's safety and continued care, and to fulfil the requirement that critical study data should be verifiable.

To achieve this, the medical records of each patient should clearly describe at least:

- That the patient is participating in the study, e.g. by including the enrolment number and the study code or other study identification;
- Date when Informed Consent was obtained from the patient and statement that patient received a copy of the signed and dated Informed Consent;
- Results of all assessments confirming a patient's eligibility for the study;
- Diseases (past and current; both the disease studied and others, as relevant);
- Results of assessments performed during the study;
- Treatments given, changes in treatments during the study and the time points for the changes;
- Serious Adverse Events (if any) including causality assessments;
- Date of, and reason for, withdrawal from study;

Ethnicity will be recorded directly into the Case Report Form (meaning that CRF is source data and not the hospital records).

A source data list will be agreed upon for each site specifying the source at a module or a variable level.

9.3 Study Monitoring

The investigator will be visited on a regular basis by the Clinical Study Monitor, who will check the following:

- Informed consent process
- Reporting of adverse events and all other safety data
- Adherence to protocol
- Maintenance of required regulatory documents
- Facilities and equipment (example: laboratory, pharmacy, ECG machine, etc…) if applicable
- Data completion on the CRFs including source data verification (SDV).

The monitor will review the relevant CRFs for accuracy and completeness and will ask the site staff to adjust any discrepancies as required.

Sponsor's representatives (e.g. monitors, auditors) and/or competent authorities will be allowed access to source data for source data verification in which case a review of those parts of the hospital records relevant to the study will be required.

9.4 Confidentiality

The investigator shall arrange for the secure retention of the patient identification and the code list. Patient files shall be kept for the maximum period of time permitted by each hospital. The study documentation (CRFs, Site File etc) shall be retained and stored during the study and for 15 years after study closure. All information concerning the study will be stored in a safe place inaccessible to unauthorized personnel.

9.5 Database management

Data management will be performed by the data management unit at the Clinical Trials Unit, Oslo University Hospital. The Data management procedures will be performed in accordance with the department's SOPs and ICH guidelines. The data management process will be described in the study specific Data Handling Plan and the study specific Data Handling Report after database closure.

Data entered into the eCRF will be validated as defined in the Data Validation Plan. Validation includes, but is not limited to, validity checks (e.g. range checks), consistency checks and customised checks (logical checks between variables to ensure that study data are accurately reported) for eCRF data and external data (e.g. laboratory data). A majority of edit checks will be triggered during data entry and will therefore facilitate efficient 'point of entry' data cleaning.

Data management personnel will perform both manual eCRF review and review of additional electronic edit checks to ensure that the data are complete, consistent and reasonable. The electronic edit checks will run continually throughout the course of the study and the issues will be reviewed manually online to determine what action needs to be taken.

Manual queries may be added to the system by clinical data management or study monitor. Clinical data managers and study monitors are able to remotely and proactively monitor the patient eCRFs to improve data quality.

All updates to queried data will be made by authorised study centre personnel only and all modifications to the database will be recorded in an audit trail. Once the queries have been resolved, eCRFs will be signed by electronic signature. Any changes to signed eCRFs will be approved and resigned by the Investigator.

Adverse events and medical history will be coded from the verbatim description (Investigator term) using the Medical Dictionary for Regulatory Activities (MedDRA).

Once the full set of eCRFs have been completed and locked, the Sponsor will authorise database lock and all electronic data will be sent to the designated statistician for analysis. Subsequent changes to the database will then be made only by written agreement.

The data will be stored in a dedicated and secured area at Oslo University Hospital. Data will be stored in a de-identified manner, where each study participant is recognisable by his/her unique trial subject number. The data will be stored until 15 years following the last patient's final study visit.

10 STATISTICAL METHODS AND DATA ANALYSIS

10.1 Determination of Sample Size

The rate of decline in SARS-CoV-2 viral load from baseline to 96 hours will be used as the basis for determination of sample size. Little is known about the variance of the viral load decline rate under standard of care and the possible efficacy of the intervention. Thus, we are planning this trial as an adaptive group sequential trial assuming a rate reduction from baseline of 6 log_{10} copies/mL/24 hours for the control group and 8 for the active group with a standard deviation of 4 within the first 96 hours after inclusion. Under these assumptions and with a Pocock type uniform alpha spending function (see **Figure 2**) with 4 looks the maximum sample size will be 202 using a 2.5% one-sided significance level to reach 90% power to detect a difference between the treatments. The interim analyses will be at 51, 101, 151 and 202 completed trial subjects. The assumptions underlying the sample size calculation will be re-evaluated at each interim analysis possibly warranting changes in the sample size without affecting the Type 1 error rate.

Figure 2.

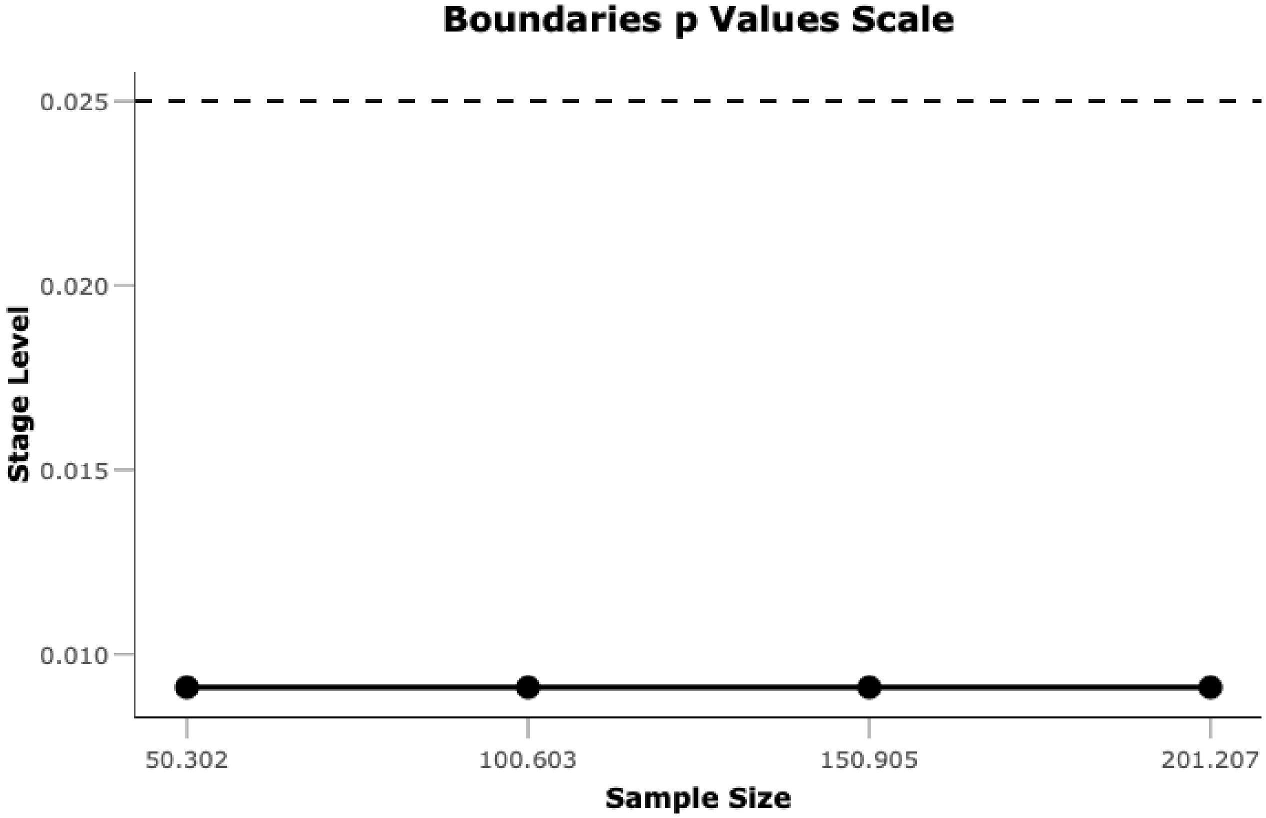

10.2 Randomization

10.2.1 Allocation- sequence generation

Eligible patients will be allocated in a 1:1 ratio, using a computer randomization procedure. The allocation sequence will be prepared by an independent statistician.

10.2.2 Allocation- procedure to randomize a patient

The computer-generated randomized allocation sequence will be imported into the eCRF system and made available to site personnel responsible for the participant enrolment. Randomization allocation will automatically be visible when enrolling a new eligible patient. This is an open-label study and no steps to conceal allocation are necessary.

The study statistician will be blinded to the randomization allocation for the writing of the statistical analysis plan (SAP). The authorisations bound to role of the study statistician in the eCRF when reading or downloading data will ensure that the statistician won't see the treatment allocation until database lock.

10.2.3 Blinding and emergency unblinding

This is an open-label study. However, the staff at the central laboratory at OUS as well as the statistician responsible for analysis of the data will be blinded to the treatment allocation for the writing of the SAP.

10.3 Population for Analysis

The following populations will be considered for the analyses:

- Intention to treat (ITT) population: All randomized participants will be included in the main ITT analyses, regardless of protocol adherence.

- Per-protocol population (PP): Includes all patients in the ITT population having completed the study treatment without major protocol violations. Criteria for inclusion in the PP population will be specified in the SAP and the final criteria will be defined prior to database lock

- Safety population: Includes all subjects with any safety information after baseline. Patients randomised to hydroxychloroquine without receiving any amount of the treatment will be excluded from the safety population.

- Total population: All enrolled participants independent of study arm will be used for additional analyses in the total population.

The primary population is the ITT population.

10.4 Planned analyses

This is a group sequential adaptive trial where analyses are planned after 51, 101, 151 and 202 completed patients.

Each statistical analysis is planned when

- The planned number of patients have been included

- All included patients have either finalized their last assessment discharge or has/is withdrawn/lost to follow-up according to protocol procedures

- All data have been entered, verified and validated according to the data management plan

Prior to each statistical analysis, the data in the data base will be exported and the exported data will be locked for further altering of data. A SAP will provide details on the planned statistical analyses. The SAP will be finalized, signed and dated prior to first interim analysis. The statistical interim analysis will be performed by an unblinded Data Monitoring Committee (DMC) statistician based on program code from the trial statistician. The trial statistician will remain blinded to treatment when finalising the SAP prior to first interim analysis and throughout the trial until final database lock. The unblinded statistician performing the analysis will only provide the study group with information on whether the trial should be stopped or continued. Details of the DMC and their procedures will be given in a separate DMC charter.

10.5 Statistical Analysis

10.5.1 Primary analysis

- The primary outcome is rate of decline in SARS-CoV-2 viral load in oropharyngeal samples as assessed by RT-PCR in samples collected at baseline, 48 and 96 hours after randomization.

The primary outcome will be analysed using a generalized linear mixed model, with subject-specific random intercept and slope, adjusted for stratification factors in the randomization.

10.5.2 Secondary analyses

Between group comparisons will be performed for the primary variable on the per-protocol population in addition to secondary efficacy endpoints on both efficacy populations (ITT and PP populations).

The between-group comparisons for secondary variables will be tested as for the primary variable where applicable and additional analyses will be performed based on the following methods (but not limited to):

- Continuous secondary variables will be subject to repeated measures mixed models or appropriate non-parametric alternatives
- Binary response variables will be analysed using logistic regression (possibly adjusting for within-subject dependencies by generalized estimating equations or mixed models) or chi-square/Mantel-Haenszel tests
- Time-to-event variables will be analysed using the Kaplan-Meier method and comparisons between the two groups will be performed using the log rank test, Cox regression analyses or appropriate parametric models such as the gamma or Weibull model.

Unless otherwise specified, all statistical hypotheses will be tested as the primary variable, i.e. with an assessment of superiority of the estimated difference between the groups. All efficacy analyses will be presented with the results from the hypothesis testing (by p-value) in addition to estimates and 95% confidence limits of the treatment effect.

10.5.3 Safety analyses

The safety analyses population will include all patients. Safety analyses will be descriptive and presented as summary tables by treatment group.

10.5.4 Descriptive statistics

Descriptive statistics will be presented with number and percentages for categorical variables, and means, standard deviation, and range for continuous variables. In case of clearly skewed continuous variables, they will be presented with median, interquartile range (25th and 75th percentiles) and range. Demographics and baseline characteristics will be presented with descriptive statistics without any hypothesis testing.

10.5.5 Missing data

If missing data is regarded as having a significant effect on the conclusions of the trial, sensitivity analyses with different methods for handling missing data will be included. Such methods may include complete case analyses, last observation carried forward, worst case/best case imputation and multiple imputation techniques.

11 STUDY MANAGEMENT

11.1 Investigator Delegation Procedure

The principal investigator is responsible for making and updating a “delegation of tasks” document listing all the involved co-workers and their role in the project. He will ensure that appropriate training relevant to the study is given to all of these staff, and that any new information of relevance to the performance of this study is forwarded to the staff involved.

11.2 Protocol Adherence

Investigators ascertain that they will apply due diligence to avoid protocol deviations. All significant protocol deviations will be recorded and reported in the Clinical Study Report (CSR).

11.3 Study Amendments

If it is necessary for the study protocol to be amended, the amendment and/or a new version of the study protocol (Amended Protocol) must be notified to and approved by the Competent Authority and the Ethics Committee according to EU and national regulations.

11.4 Audit and Inspections

Authorized representatives of a Competent Authority and Ethics Committee will visit the centre to perform inspections, including source data verification. Likewise the representatives from sponsor may visit the center to perform an audit. The purpose of an audit or inspection is to systematically and independently examine all study-related activities and documents to determine whether these activities were conducted, and data were recorded, analysed, and accurately reported according to the protocol, Good Clinical Practice (ICH GCP), and any applicable regulatory requirements. The principal investigator will ensure that the inspectors and auditors will be provided with access to source data/documents.

12 ETHICAL AND REGULATORY REQUIREMENTS

The study will be conducted in accordance with ethical principles that have their origin in the Declaration of Helsinki and are consistent with ICH/Good Clinical Practice and applicable regulatory requirements. Registration of patient data will be carried out in accordance with national personal data laws.

12.1 Ethics Committee Approval

The study protocol, including the patient information and informed consent form to be used, must be approved by the regional ethics committee before enrolment of any patients into the study.

12.2 Other Regulatory Approvals

The protocol will be submitted and approved by the applicable competent authorities before commencement of the study. Amendments to the protocol will be submitted to the competent authorities according to local regulations.

The protocol will be registered in www.clinicaltrials.gov before inclusion of the first patient.

Application to the Norwegian Medicines Agency will be approved before inclusion of the first patient.

Collection, storage and analyses of all data and sensitive information will be conducted according to current General Data Protection Regulation (GDPR) and in accordance with approval from the local Data Protection Official.

12.3 Informed Consent Procedure

The investigator is responsible for giving the patients full and adequate verbal and written information about the nature, purpose, possible risk and benefit of the study. They will be informed as to the strict confidentiality of their patient data, but that their medical records may be reviewed for trial purposes by authorized individuals other than their treating physician.

It will be emphasized that the participation is voluntary and that the patient is allowed to refuse further participation in the protocol whenever she/he wants. This will not prejudice the patient's subsequent care. Documented informed consent must be obtained for all patients included in the study before they are registered in the study. This will be done in accordance with the national and local regulatory requirements. The investigator is responsible for obtaining signed informed consent.

A copy of the patient information and consent will be given to the patients. The signed and dated patient consent forms will be filed in the Investigator Site File binder.

In cases where the patient does not speak Norwegian, a professional translator will be used.

12.4 Subject Identification

The investigator is responsible for keeping a list of all patients (who have stopped treatment or undergone any study specific procedure) including patient's date of birth and personal number, full names and last known addresses.

The patients will be identified in the CRFs by patient number.

13 TRIAL SPONSORSHIP AND FINANCING

The study is sponsored by Akershus University Hospital.

14 TRIAL INSURANCE

The Principal investigator has insurance coverage for this study through membership of the Drug Liability Association (see http://www.laf.no for more details).

15 PUBLICATION POLICY

Upon study completion and finalization of the study report the results of this study will either be submitted for publication and/or posted in a publicly assessable database of clinical study results.

The results of this study will also be submitted to the Competent Authority and the Ethics Committee according to EU and national regulations.

All personnel who have contributed significantly with the planning and performance of the study (Vancouver convention 1988) may be included in the list of authors.

16 REFERENCES

1. Ren LL, et al. Identification of a novel coronavirus causing severe pneumonia in human: A descriptive study. Chin Med J (Engl) 2020;Publish Ahead of Print.
2. WHO. Coronavirus disease (covid-19) outbreak. https://www.who.int/emergencies/diseases/novel-coronavirus-2019 (Accessed March 12 2020).
3. WHO. R&d blueprint. Informal consultation on prioritization of candidate therapeutic agents for use in novel coronavirus 2019 infection. 2020.
4. Wang M, et al. Remdesivir and chloroquine effectively inhibit the recently emerged novel coronavirus (2019-ncov) in vitro. Cell Res 2020;30:269-71.
5. Gao J, et al. Breakthrough: Chloroquine phosphate has shown apparent efficacy in treatment of covid-19 associated pneumonia in clinical studies. Bioscience trends 2020:10.5582/bst.2020.01047.
6. Multicenter Collaboration Group of Department of Science Technology of Guangdong Province Health Commission of Guangdong Province for Chloroquine in the Treatment of Novel Coronavirus Pneumonia. [expert consensus on chloroquine phosphate for the treatment of novel coronavirus pneumonia]. Zhonghua jie he he hu xi za zhi = Zhonghua jiehe he huxi zazhi = Chinese journal of tuberculosis and respiratory diseases 2020;43:E019.
7. Ison MG, et al. End points for testing influenza antiviral treatments for patients at high risk of severe and life-threatening disease. The Journal of infectious diseases 2010;201:1654-62.
8. McGinley A, et al. A national early warning score for acutely ill patients. BMJ 2012;345:e5310.
9. Zhou F, et al. Clinical course and risk factors for mortality of adult inpatients with covid-19 in wuhan, china: A retrospective cohort study. Lancet 2020.
10. Yao X, et al. In vitro antiviral activity and projection of optimized dosing design of hydroxychloroquine for the treatment of severe acute respiratory syndrome coronavirus 2 (sars-cov-2). Clin Infect Dis 2020.
11. Trinkley KE, et al. Qt interval prolongation and the risk of torsades de pointes: Essentials for clinicians. Curr Med Res Opin 2013;29:1719-26.
12. Zou L, et al. Sars-cov-2 viral load in upper respiratory specimens of infected patients. N Engl J Med 2020.

17 LIST OF APPENDICES

Summary of Product Characteristics “PLAQUENIL”
